# *Ex vivo* Live Cell Imaging of Nanoparticle-Cell Interactions in the Mouse Lung

**DOI:** 10.3389/fbioe.2020.588922

**Published:** 2020-10-30

**Authors:** Fernanda Ramos-Gomes, Nathalia Ferreira, Alexander Kraupner, Frauke Alves, M. Andrea Markus

**Affiliations:** ^1^Translational Molecular Imaging, Max-Planck-Institute for Experimental Medicine, Göttingen, Germany; ^2^nanoPET Pharma GmbH, Berlin, Germany; ^3^Clinic of Hematology and Medical Oncology/Institute of Diagnostic and Interventional Radiology, University Medical Center Göttingen, Göttingen, Germany

**Keywords:** nanoparticles, live cell imaging, fluorescence microscopy, cancer, experimental lung metastasis, phagocytosis, extracellular vesicles, cell dynamics

## Abstract

A successful clinical translation of novel nanoparticle-based cancer therapeutics requires a thorough preclinical investigation of their interaction with immune, tumor and endothelial cells as well as components of the tumor-microenvironment. Although high-resolution microscopy images of fixed tumor tissue specimens can provide valuable information in this regard, they are only static snapshots of a momentary event. Here we describe a superior alternative fluorescence microscopy approach to assess the feasibility of investigating nanoparticle-cell interactions in the mouse lung live and over time at nanometer resolution. We applied fluorescent lung tumor cells and Barium-based fluorescently labeled nanoparticles to nude mice or to CD68-EGFP transgenic mice for visualization of the monocyte-macrophage lineage. Shortly before imaging, fluorescently labeled lectin was intravenously injected for staining of the blood vessels. The lung was filled *ex vivo* with 1% agarose and individual lung lobes were imaged over time using a confocal microscope with Airyscan technology. Time series demonstrate that live cell imaging of lung lobes can be performed for at least 4 h post mortem. Time-lapse movies illustrate the dynamics of the nanoparticles within the pulmonary circulation and their uptake by immune cells. Moreover, the exchange of nanoparticle material between cancer cells was observed over time. Fluorescent monocytes in lungs of CD68-EGFP transgenic mice could be visualized within blood vessels in the process of interaction with tumor cells and nanoparticles. This high resolution *ex vivo* live cell imaging approach provides an excellent 4D tool to obtain valuable information on the behavior of tumor and immune cells at first encounter with nanoparticles and may contribute to the understanding of how nanoparticles interact with cells supporting the development of therapeutic strategies based on nanoparticulate drug delivery systems.

## Introduction

Routinely used chemotherapy has a number of weaknesses, including poor pharmacokinetics, low specificity and off target effects, leading to substantial side-effects. Nanoparticle (NP)-based cancer therapeutics have therefore been increasingly explored, because NPs have a number of advantages compared to “naked” therapies: (i) improved drug solubility and stability, (ii) prolonged drug half-live in plasma, (iii) minimized off-target effects, (iv) improved accumulation of drugs at a target site due to the possibility of functionalization and (v) better controlled concentration of drugs by packaging in NPs. Furthermore, recent efforts allowed the incorporation of therapeutic agents into biocompatible NPs, therefore, reducing undesirable local or systemic effects (Li et al., 2012). Combining all these characteristics, some NPs have been successfully approved by the FDA or are currently being tested in clinical trials (Ventola, 2017). However, most NP-based cancer drugs have either not made it into the clinic or have failed to significantly improve patient outcome. This is most likely due to the inter-and intra-individual heterogeneity of tumors and to the complexity of tumor and immune response that has been underestimated. For instance, the Enhanced Permeability and Retention (EPR) effect, was widely believed to be a general and homogenous feature of most tumors, a phenomenon caused by leaky vessels by which nanodrugs would predominantly accumulate in the tumor. It has now been recognized that the EPR effect is greatly influenced by a number of parameters, including the tumor microenvironment, vessel density and permeability and stroma composition (Dasgupta et al., 2020).

A further deciding factor for the success of nanotherapies, in particular nanoparticulate immunotherapies, is the specific immune response induced by these (Khalil et al., 2016). As Blank et al. recently summarized, modulation of immune response progresses in different steps of the immune cell-antigen interaction, comprising antigen uptake, trafficking, processing and presentation to T cells. These steps require thorough analysis, also as part of pharmacologic and biocompatibility testing in the development process of novel NP-based cancer therapeutic strategies. The safe design of nanotherapies should therefore include the careful characterization of the interaction of NPs with immune cells, tumor cells, the microenvironment and endothelial cells (blood vessels) (Blank et al., 2017). Moreover, a thorough assessment of the targeted accumulation of nanoparticulate drugs at the desired site is crucial, as the EPR effect is not a reliable feature of all tumors.

To avoid side effects upon systemic delivery of therapies, local applications of nanoparticulate therapies have been already tested, for instance by intratumoral injection (Marabelle et al., 2014) or in the case of lung cancer by inhalation (Liu et al., 2019). A rapid uptake of NPs by pulmonary antigen-presenting cells (APCs) has been repeatedly demonstrated and the targeted delivery of immunostimulants to intratumoral APCs in the lung has recently been shown (Liu et al., 2019). Studies *in vitro* and *in vivo* further suggested both dendritic cells (DCs) and T cells as promising targets for pulmonary NP therapies (Nembrini et al., 2011; Blank et al., 2017; Jia et al., 2018). The lung thus seems to be an ideal organ for specific delivery of nanoparticulate drugs as the relevant immune cells can be directly targeted by inhalation and intravenous application also results in deposition in the lung. A particular close look should be taken at the interaction of NPs with the monocyte/macrophage lineage, which plays a key role in both innate adaptive immune response and is involved in all common pulmonary diseases, including allergic asthma, chronic obstructive pulmonary dysplasia (COPD) and lung cancer (Arora et al., 2017). Tumor associated macrophages (TAMs) not only play a key role in tumor therapy response (Cassetta and Kitamura, 2018; Rodell et al., 2018), but have also been successfully used as theranostic targets for the combined treatment and imaging of lung tumors (Markus et al., 2015; Cuccarese et al., 2017; Napp et al., 2018).

Imaging of the numerous processes and interactions of NPs with lung immune cells, microenvironment and/or tumor cells and the elimination of NPs from the lung has traditionally been done by histology and immunohistochemistry. While these microscopic methods provide cellular nanoscale resolution, they are only static snapshots of momentary events and cannot provide the same information as live cell imaging. Other live cell or *in vivo* technologies, such as near infrared fluorescence optical imaging, computed tomography (CT), magnetic resonance imaging (MRI), and positron emission tomography (PET) or ultrasound do not provide the resolution to image at the cellular or nanomaterial resolution.

Here we demonstrate an alternative technique for monitoring the live interaction of monocytes/macrophages and tumor cells with NPs in the lung. We show that *ex vivo* live cell confocal microscopy of entire mouse lung lobes provides an excellent 4D tool for imaging of several dynamic processes in tumor tissue, such as the traffic of cells, shedding of extracellular vesicles (EVs) and the accumulation of NPs in tumor tissue.

## Methods and Materials

### Materials

Barium sulfate (BaSO4)-based nanoparticles (Ba-NPs) were produced by chemical precipitation at ambient conditions. After a purification step the particles were sterically stabilized using a biocompatible polymer. The so obtained highly stable colloidal suspension was sterilized and formulated for *in vitro*/*in vivo* use. The hydrodynamic diameter of the particles was around 120 nm. Fluorescent labeling of Ba-NPs was performed via EDC/NHS coupling chemistry of amino-functionalized Atto488 (Atto-Tec GmbH, Siegen) or Cy3 fluorescent dyes to COOH groups of the stabilizing polymer. Subsequently, the labeled particles were dialyzed against water in order to remove non-bounded dye. The Ba-NPs were tested *in vitro* and were found to be non-toxic and had no effect on the vitality of mammalian cells.

Lectin-Alexa647 (Alexa647 labeled Isolectin B4 from Bandeiraea simplicifolia) was a kind gift from Roche Pharma, Penzberg.

### Cell Culture

A cell proliferation assay (MTT-assay) was performed using the MCF-7 in order to assess the cytotoxicity of the Ba-NPs. The experiments (*n* = 5 per test series) were conducted at 4 h and 24 h incubation time and with a final Ba-NP concentration of 15–45 mg/ml. The results were compared against a positive (100% death cells) and negative control (100% vital cells). The same experiments were performed with A549 cells with a Ba-NPs concentration of 30 mg/mL and 24 h incubation time.

Human A549-mCherry lung tumor cells (a kind gift from Dr. Winkler, German Primate Centre, Germany) and murine LL/2-red fluorescence protein (RFP) (Lewis Lung) cancer cells (a kind gift from Prof. Augustin, Deutsches Krebsforschungszentrum, Heidelberg) were kept at 37°C and 5% CO_2_ atmosphere. A549-mCherry cells were grown in DMEM high glucose (Gibco) supplemented with 10% fetal calf serum (FCS) (Gibco). LL/2-RFP cells were grown in DMEM high glucose supplemented with 1% penicillin/streptomycin, 1% non-essential amino acids (Gibco) and 10% FCS. When indicated, 1 × 10^6^ A549-mCherry cells were incubated with 13 mg/ml Ba-Atto488 NPs overnight, washed of excess NPs the next morning and run over a 40 μm cell strainer (Corning) before application to the mice.

### Animal Experiments

All animal *in-vivo* procedures were performed in compliance with the guidelines of the European Directive (2010/63/EU) and the German ethical laws and were approved by the administration of Lower Saxony, Germany.

Pathogen-free male NMRI-Fox1nu/nu mice, 6–8 weeks of age were purchased from Charles River Laboratories Inc. C57BL/6-Tg(CD68-EGFP)1Drg/J were originally purchased from The Jackson Laboratory and then bred in house. All animals were housed in a controlled environment with a regular 12 h dark:light cycle, at 22°C and were fed laboratory chow and tap water *ad libitum*.

Healthy mice were injected intravenously (i.v.) either with 1 × 10^6^ LL/2-RFP cells or 1 × 10^6^ Ba-Atto488-NP-loaded A549-mCherry cells or with 6 mg Ba-Cy3 NPs, 5 min before sacrifice.

Experimental lung metastases were obtained by i.v. injection of 1 × 10^6^ A549-mCherry cells into male NMRI-Fox1^nu/nu^ mice. The development of lung metastasis was assessed by microCT (Quantum FX, Perkin Elmer) every other week. Once lung metastasis were visible (about 8 weeks after induction), 6 mg Ba-Atto488 NPs were injected i.v. 5 min before sacrifice.

All mice were i.v. injected with 100 μg Lectin-Alexa647 shortly before sacrifice.

### Preparation of Lungs for Live Cell Imaging

The lungs were prepared as described previously with some modifications (van den Bijgaart et al., 2016). Briefly, mice were sacrificed by isoflurane overdose and cervical dislocation and the trachea exposed. Following a small incision at the top of the trachea, a blunt cannula (20G, the tips cut-off) was inserted max. 1 cm into the trachea and fixed with common cotton thread. The lungs were immediately filled with 600 μl of 37°C warm 1% agarose (BioFroxx) in DMEM w/o phenol red (Gibco 31053-028). The agarose filled lungs were tied with a cotton thread at the trachea underneath the inserted cannula to prevent leaking of the agarose before setting and were then dissected from the mouse. Individual lung lobes were then placed on uncoated ibidi 35 mm cell culture dishes (ibidi GmbH) with the flat side down, covered entirely with warm 1% agarose-DMEM and imaged by confocal microscopy as soon as the agarose was set.

### Confocal Microscopy

A Zeiss LSM880 confocal laser scanning microscope (CLSM, Carl Zeiss Microscopy GmbH) was used, equipped with an Airyscan detection unit and GaAsP-PMT/Spectral detectors, a 20 × air objective lens (Plan-APOCHROMAT, NA: 0.45, air, DIC), a motorized stage, incubator for live cell conditions and a tuneable laser (470–670 nm). mCherry and Red Fluorescence Protein (RFP) were excited at 561 nm, Ba-Atto488 NPs and CD68-EGFP were excited at 488 nm and Lectin-Alexa647 was excited at 633 nm. The experiments were performed under live cell environmental conditions (37°C/5% CO_2_) and the whole equipment was turned on the evening before imaging to minimize tissue movement due to temperature changes.

### Light Sheet Microscopy

Following confocal microscopy the same lung lobes were fixed in 4% paraformaldehyde (PFA) overnight and then cleared using the ethyl-3-phenylprop-2-enoate (ethyl cinnamate, ECi) protocol described before (Klingberg et al., 2017). Images were acquired with an UltraMicroscope II (LaVision BioTec) with an Olympus MVX10 Zoom Microscope Body (Olympus,), a white light laser module, an Andor Neo sCMOS camera, and detection optics with an optical magnification range from 1.26 × to 12.6 × and an NA of 0.6 were used. The following filter settings were used for excitation/emission of: EGFP 520 ± 40/585 ± 40 nm; mCherry 560 ± 40/620 ± 60 nm and LectinA647 630 ± 30/680 ± 30 nm. Z-step size was set to 20 μm and a 2 × optical zoom factor was used. Stitched 3D mosaics were composed of 8 tiles of 215 Z-stacks in a total range of 4,280 μm.

### Histology

Following light sheet microscopy the same lung lobe was paraffin embedded and 2 μm sections were cut using a Leica EG 1150C Microtome. Deparaffinized sections were stained with Haemotoxylin and Eosin (H&E) and imaged using an Axiovert 200 M inverted microscope (Carl Zeiss Microscopy GmbH).

### Image Analysis

All images, including volumetric 3D images of microscopy z-stacks, were processed and analyzed with the software Imaris 9.1.2 (Bitplane), Imaris Stitcher, FIJI (an image-processing package based on ImageJ) and Graph Pad Prism 7.05 (Graph Pad Software, Inc.). Maximum 3D projections of z-stacks are presented. Videos were produced from time series using Imaris 9.1.2.

## Results and Discussion

### Set-Up of *ex vivo* Live Cell Imaging of the Mouse Lung

To demonstrate the feasibility of *ex vivo* live cell imaging we chose three biological scenarios in the mouse lung:

(i) To evaluate the immediate interaction of NPs and tumor cells with the immune cells and the surrounding lung tissue we either i.v. injected LL/2-RFP cells (Bertram and Janik, 1980) or fluorescently labeled Ba-NPs (Ba-Atto488 or Ba-Cy3) in CD68-EGFP or Nu/Nu mice.

(ii) To evaluate the inter-cellular behavior of tumor cells, we incubated A549-mCherry cells with Ba-Atto488 NPs overnight and injected them i.v. into Nu/Nu mice.

(iii) To visualize the tumor cell dynamics in the tumor mass and the interactions of NPs with tumor tissue we used the model of experimental lung metastasis.

The use of the fluorescent protein-expressing-cancer cells (A549-mCherry and LL/2-RFP), transgenic fluorescent protein expressing animals (CD68-EGFP), and fluorescent tags (Lectin-A647 and Ba-NPs) enabled the easy discrimination of tumor cells, tumor masses, blood vessels and NPs and their specific interactions (Hoffman, 2009). The human tumor cell line A549 is commonly used for experimental metastasis and produced solid nodules in the lungs of Nu/Nu mice after i.v. application, as previously described (Liu et al., 2012).

As an illustrative example for NPs we chose Ba-based NPs of about 120 nm in size that are non-toxic. Cell proliferation tests (MTT assay) at 4 h incubation showed no cytotoxic effects over the tested concentration range while incubation at 24 h time showed a concentration dependent trend, but still no relevant cytotoxic effects (cell vitality > 80 %) (Supplementary Figure 1). MTT assay with A549 cells at 24 h incubation time showed identical results (data not shown). Ba-NPs were either labeled with Atto488 or Cy3 fluorescent dyes that resulted in a detectable resolution range due to their formation of small agglomerates. Furthermore, they were efficiently taken up by macrophage/monocytes and tumor cells *in vitro* (data not shown).

Blood vessels were stained *in vivo* with the widely used protein Lectin, which is known to bind to glycoproteins located in the glycocalyx and in the basal membrane of endothelial cells. The visualization of the blood vessels provided a good “counterstain” of the lung tissue structure and excellent means to monitor the stability of the tissue over the time measurements.

We chose to sacrifice the animals shortly after injection of NPs because we were interested in visualizing the immediate reaction of tumor and immune cells to NPs in the lung. The filling of the lungs with agarose following the sacrifice of the mice prevented the collapse of the lungs and maintained the structural integrity of the lung. Environmental control settings during the image acquisition process provided physiological conditions to maximize the length of cell viability within the explanted lung. The total process from i.v. injection of NPs or tumor cells to acquisition of the first image took on average 40–60 min.

Combined, this *ex vivo* imaging approach allowed us to assess several live cell processes in the lung tissue at cellular resolution and up to a time period of at least 4 h. These findings are in accordance with results previously described by van den Bijgaart et al. (2016). While they showed the suitability of the technique to monitor the activity of metastatic and immune cells over a similar time period, we were able to additionally demonstrate the interaction with NPs and shedding of extracellular vesicles (EVs) at a substantially improved resolution. Most importantly, we were able to demonstrate cell dynamics and motility at quantifiable numbers by assuring minimal tissue movement during acquisition.

Live cell imaging of the mouse lung has been attempted before by intravital lung imaging using intercostal windows, however this is a very complicated technique and stressful for the animal (Headley et al., 2016). Other imaging approaches such as endoscopy or fluorescence reflectance imaging provide significantly lower resolution. The *ex vivo* live cell imaging method presented here also requires no *ex vivo* staining procedures if fluorescently labeled nanoparticles and fluorescently tagged cell lines are used for the induction of tumors. The approach described here thus provides a powerful 4D tool for monitoring nanoparticle-cell and cell-cell interactions.

In the following we present several scenarios where this technique has proven to be useful for *ex vivo* live cell imaging of the lung.

### Reaction of Macrophages and Monocytes Toward Tumor Cells

The i.v. injection of tumor cells is a common method to mimic circulating tumor cells (CTCs) which can be generated by primary tumors and can lead to metastasis. From the circulation, the cells reach the lung within minutes. We therefore i.v. injected LL/2-RFP cells into CD68-EGFP transgenic mice to examine the behavior of tumor cells at the immediate contact with immune cells.

**Figure 1:**
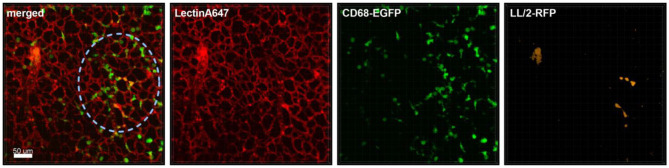
Monocyte recruitment and surveillance of tumor cells within the lung. Representative confocal images of a lung lobe from a CD68-EGFP mouse (macrophages and monocytes green), showing the macrophages/monocytes distribution in the lung parenchyma/vessels visualized by staining of blood vessels with Lectin-A647 (red) in close proximity to LL/2-RFP tumor cells (yellow). Tumor cells and Lectin-A647 were applied shortly before sacrifice. Images show the overlay (merged) of Lectin-A647 (red), CD68-EGFP cells (green), and LL/2-RFP cells (yellow). Scale bar represents 50 μm.

In the first hour of acquisition we could observe the recruitment of macrophages/monocytes to the tumor cell-rich regions and their surveillance (Figure 1). Supplementary Movie 1A, which includes the Lectin-A647 stained blood vessel imaging, shows minimal movement of the lung tissue and high focus stability over a period of 3 h 20 min. Moreover, Supplementary Movie 1B shows that the interaction of monocytes and macrophages with the tumor cells is a very immediate process, where within 5 min frame intervals we observed immune cells which directly contacted the tumor cells and appeared and disappeared from the video frame. Furthermore, Supplementary Movie 1B illustrates that tumor cells produce extensive extracellular vesicles (EVs), which are partly captured by CD68-positive cells (white arrow). Blebbing of cells was also observed at about 2 h of acquisition, suggesting apoptotic processes of tumor cells around 3 h after their arrival in the lung tissue. Moreover, the phagocytosis of tumor cells by macrophages/monocytes is seen in Supplementary Movie 1B (yellow arrow).

**Figure 2:**
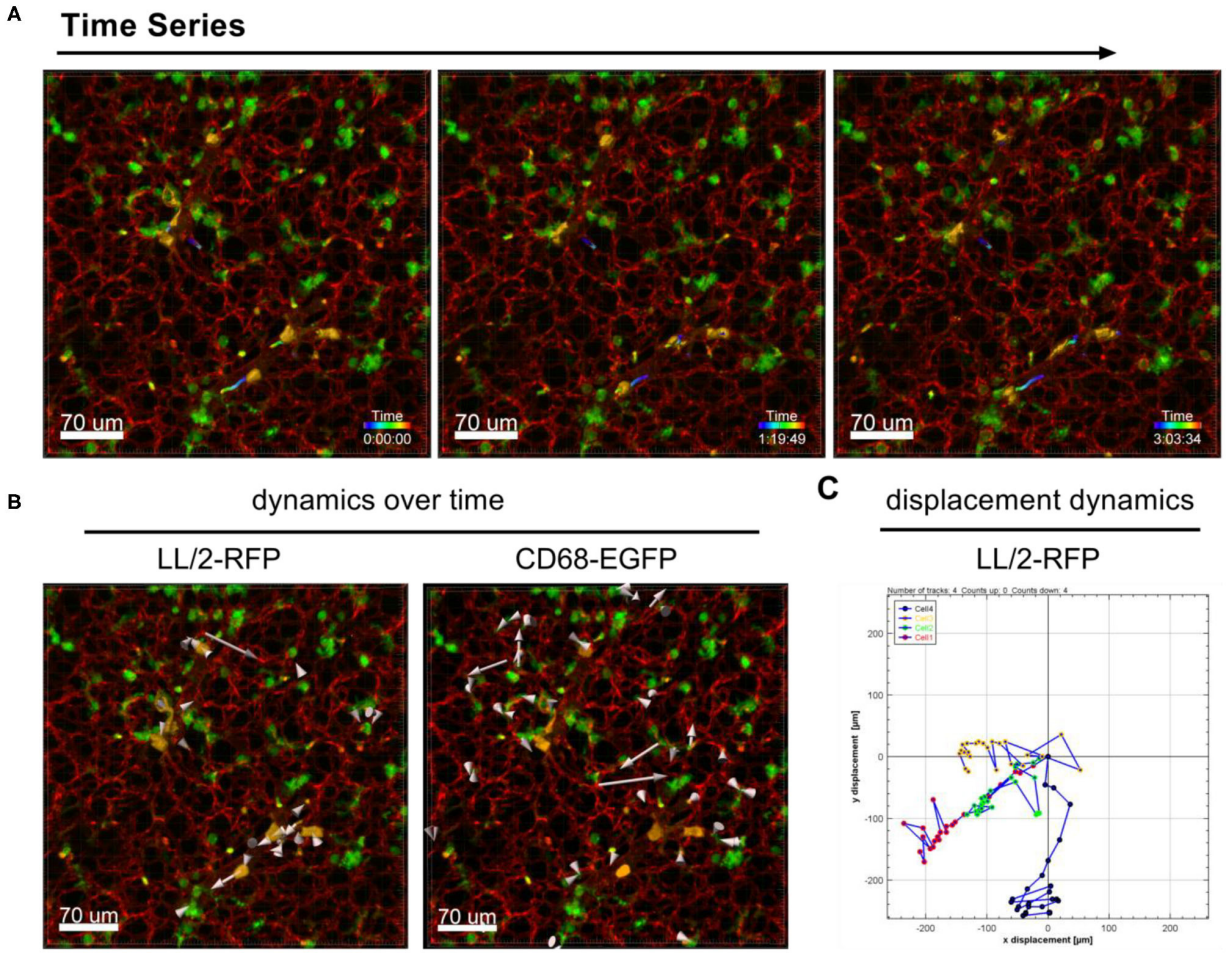
Tracking of cell dynamics. Confocal images of a lung lobe from a CD68-EGFP mouse (macrophages and monocytes green), injected with Lectin-A647 (red), and LL/2-RFP cells (yellow) i.v. 5 min before sacrifice. **(A)** Representative frames of a time series over 3 h at an interval of 300 s showing the trajectories of LL/2-RFP cells within blood vessels. **(B)** Two representative examples of how individual cells can be tracked over time. Arrow length represents the distance of displacement of individual cells over time. **(C)** Exemplary measurement of the 2D displacement dynamic of 4 LL/2-RFP cells. Scale bars represent 70 μm.

The dynamic movement of monocytes/macrophages and tumor cells were tracked over time in lungs of CD68-EGFP mice which received an i.v. injection of LL/2-RFP tumor cells as shown in Figure 2A and Supplementary Movie 2. The displacement dynamics of both cell types were recorded over 87 min (Figure 2B) and were quantified in Figure 2C.

No other cell is more appropriate than the macrophage to show the need that cells have to be examined in their natural setting. Only recently, Hussel and Bell stressed in a review that alveolar macrophages exist in a unique microenvironment of the airway lumen which can have a considerable influence on many aspects of their phenotype, function and turnover (Hussell and Bell, 2014). There is for instance little understanding of the extent to which alveolar macrophages interact with the epithelium, blood vessels or with one another in their response to pathogens, allergens, or environmental challenges. Additionally, different macrophage lineages, for example tissue-resident and monocyte-derived macrophages, may respond differently to cytokines and other signals received from tumor cells (Ham et al., 2020).

However, our knowledge on TAMs, monocytes and the interaction of these with tumor cells, microenvironment mainly comes from *in vitro* and histological examinations, leaving a large information gap on how these cell lineages behave *in vivo*. Recently, Headley et al. used intravital microscopy using an intercostal window to describe the reaction of macrophages to freshly arrived CTCs in the lung tissue (Headley et al., 2016). Their results show the dynamic generation of tumor microparticles by the CTCs in capillaries and the loading of such material onto cells of the myeloid lineage. Our *ex vivo* live cell imaging approach confirms such findings using a much simpler technique and demonstrates the feasibility to shed some light on some of the processes involving the monocyte/macrophage lineage and the behavior of CTCs.

### Reaction of Macrophages and Monocytes Toward Nanoparticles

TAMs and monocytes are also important for the specific accumulation of NPs, which are increasingly explored for therapeutic drug delivery in oncology (Cuccarese et al., 2017). Nanomedicines can also be engineered to inhibit the recruitment, kill or re-educate TAMs, and imaging TAMs with NPs can support diagnosis and prognosis of cancer (Andón et al., 2017).

**Figure 3:**
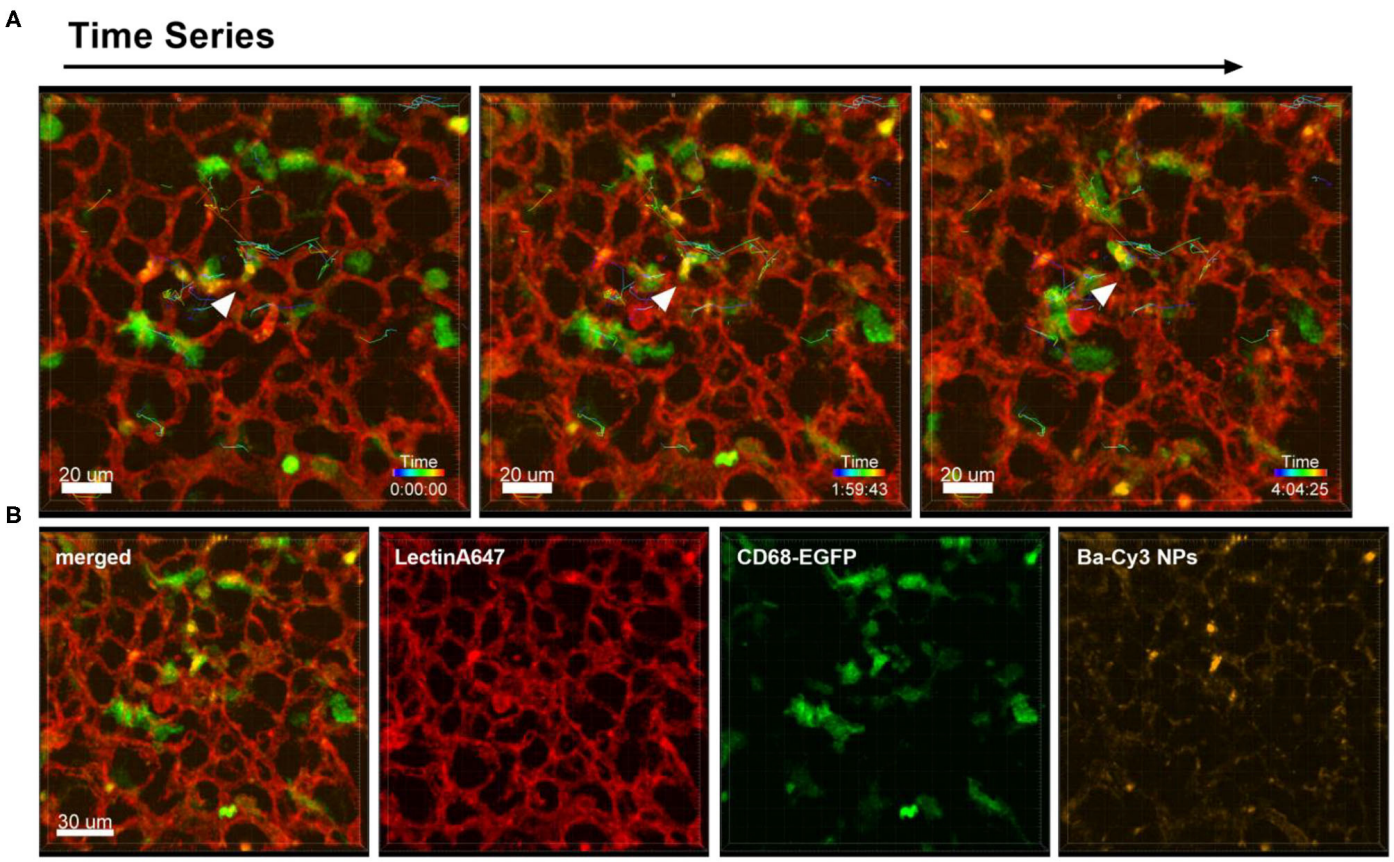
Uptake of NPs by monocytes and their tracking. Representative confocal images of a lung lobe from a CD68-EGFP mouse (macrophages and monocytes green). Lectin-A647 for visualization of blood vessels (red) and Ba-Cy3 NPs (yellow) were injected i.v. 5 min before sacrifice. **(A)** Representative frames of a time series over 4 h at an interval of 300 s showing the trajectory of individual monocytes. Scale bar represents 20 μm. **(B)** shows the co-localization of the Ba-Cy3 NPs with the CD68-EGFP positive monocytes within blood vessels. Images show the overlay (merged) of Lectin-A647 (red), CD68-EGFP cells (green), and Ba-Cy3 NPs (yellow). Scale bar represents 30 μm.

Different to the behavior of immune cells toward tumor cells described above, the i.v. injection of NPs into healthy CD68- EGFP mice did not produce the same recruitment effect of blood monocytes and alveolar macrophages, since the NPs have a more homogenous dispersion throughout the lung tissue. Figure 3 and Supplementary Movie 3 are produced from lung lobes of CD68-EGFP mice that received an i.v. injection of Ba-Cy3 NPs. The images show that CD68 positive blood monocytes (green) which appear smaller than the also CD68-positive alveolar macrophages, are excellent phagocytes, internalizing the Ba-Cy3 NPs and possibly processing them (Figure 3B). Figure 3A and Supplementary Movie 3 also show how active the Ba-Cy3-NP-loaded monocytes move up and down the blood vessels, which can be tracked over time.

A limitation of the technique is the necessity to use agarose to fill the lung in order to prevent collapsing of the lung. This evidently implies a somewhat unphysiological environment in the bronchial and alveolar spaces and most certainly represents a barrier for alveolar macrophages and their mobility. Processes that occur in these spaces can therefore only be observed in limits. Especially the phagocytosis of NPs and tumor cells by alveolar macrophages are likely to be hampered. We have attempted to image *ex vivo* lungs which we filled with air via the trachea and immediately enclosed in 1% agarose on a microscopy chamber to mimic the outside pressure of the pleural space and rib cage and thus prevent loss of air. Visualization of live cells, including alveolar macrophages is still possible up to 1 h post preparation, but the instability of the lung then causes movement and consequently imaging artifacts (data not shown). The described technique using agarose to fill the lung is thus mostly suited for visualization of processes that occur within the blood and lymphatic vessels and interaction of NPs with blood residing immune cells as well as endothelial cells. However, some aspects such as the induction of cell apoptosis by drugs or the internalization of NPs by alveolar macrophages may well be studied by including the drugs or NPs in the agarose. We included Ba-Cy3 NPs in the agarose and could indeed detect the phagocytosis of NPs by macrophages (data not shown).

**Figure 4:**
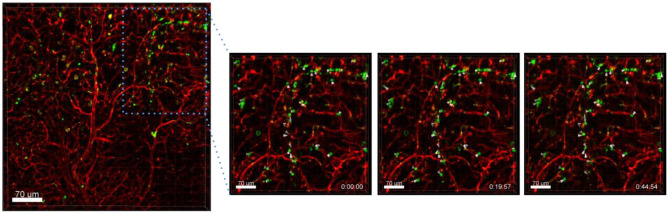
Drainage of Ba-488 NPs by lymphatic vessels. Confocal microscopy images of lung lobes which received i.v. injected Ba-Atto488 NPs (green) and Lectin-A647 (red) 30 min before sacrifice of the mouse. Left: overview image. Right: representative higher magnification images of a time series over 45 min of a selected area tracking NPs in a non-lectin-stained vessel. Scale bars represent 70 μm.

Another interesting finding was that Ba-based NPs injected i.v. into Nu/Nu mice were mostly detected in blood vessels, but some were also found moving in lectin-unstained vessels, suggestive of lymphatic channel drainage (Figure 4). For the use of NPs as drug delivery systems, the understanding of their clearance is very important. NPs can be transported to the lymph nodes through the lymphatic vessels where they could accumulate or also be transported back to the blood stream and end up in the liver or kidneys for elimination, depending on their size. NPs can also be designed to be favorably delivered to immune-rich organs such as lymph nodes or spleens (Reddy et al., 2007). In future, *ex vivo* live cell imaging may thus be used to test whether nanovaccines can be efficiently drained into the lymphatic system, enabling accumulation in lymph nodes which contain a high number of immune cells and which coordinate diverse immunomodulation events. This method may be also used to test the size dependency of NPs for lymphatic drainage of NPs. Reddy et al. reported for instance that after intradermal injection, interstitial flow transported 25 nm small nanoparticles highly efficiently into lymphatic capillaries and their draining lymph nodes, targeting half of the lymph node–residing dendritic cells, whereas 100 nm NPs were only 10% as efficient (Reddy et al., 2007).

### Delivery of Nanoparticles to the Tumor

Targeting NP-based therapies to the tumor site is obviously crucial for specific and effective treatment. It is therefore important to assess the capability of nanoparticulate drugs to reach the tumor, as we show in the following examples.

**Figure 5:**
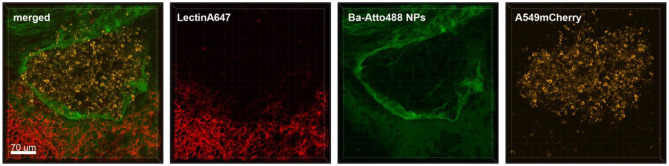
Ba-NPs barely penetrate the tumor capsule due to poor tumor vascularization. Confocal images of a lung lobe from a mouse with A549-mCherry induced lung tumor nodules that received Ba-Atto488 NPs i.v. shortly before sacrifice. Images show the overlay (merged) of Lectin-A647 for blood vessel staining (red), Ba-Atto488 NPs (green), and A549-mCherry positive nodules (yellow). Scale bar represents 70 μm.

Experimental lung metastasis was obtained by i.v. injection of A549-mCherry cells in NMRI-Fox1nu/nu mice that led to the formation of tumor nodules in the lung that were easily detectable by their fluorescence (Figure 5). Blood vessel staining of the nodules revealed that they are poorly perfused and possess a strong connective tissue capsule (Figure 5).

Although NPs have progressively been used as drug delivery systems their successful transport to the tumor lesion or TAMs is not always a given. We tested the feasibility of *ex vivo* lung live cell imaging for assessing the penetration and accumulation of Ba-NPs at the tumor site. Figure 5 shows a lung lobe of a mouse with an A549-mCherry positive lung metastasis which received an i.v. injection of Ba-Atto488 NPs. It is clearly visible that the type and size of NPs used here strike a barrier around the tumor mass that they cannot penetrate and only a very small proportion of NPs reach the core of the tumor, due to the limited vascularization of the nodule, as illustrated by the lack of lectin staining.

**Figure 6:**
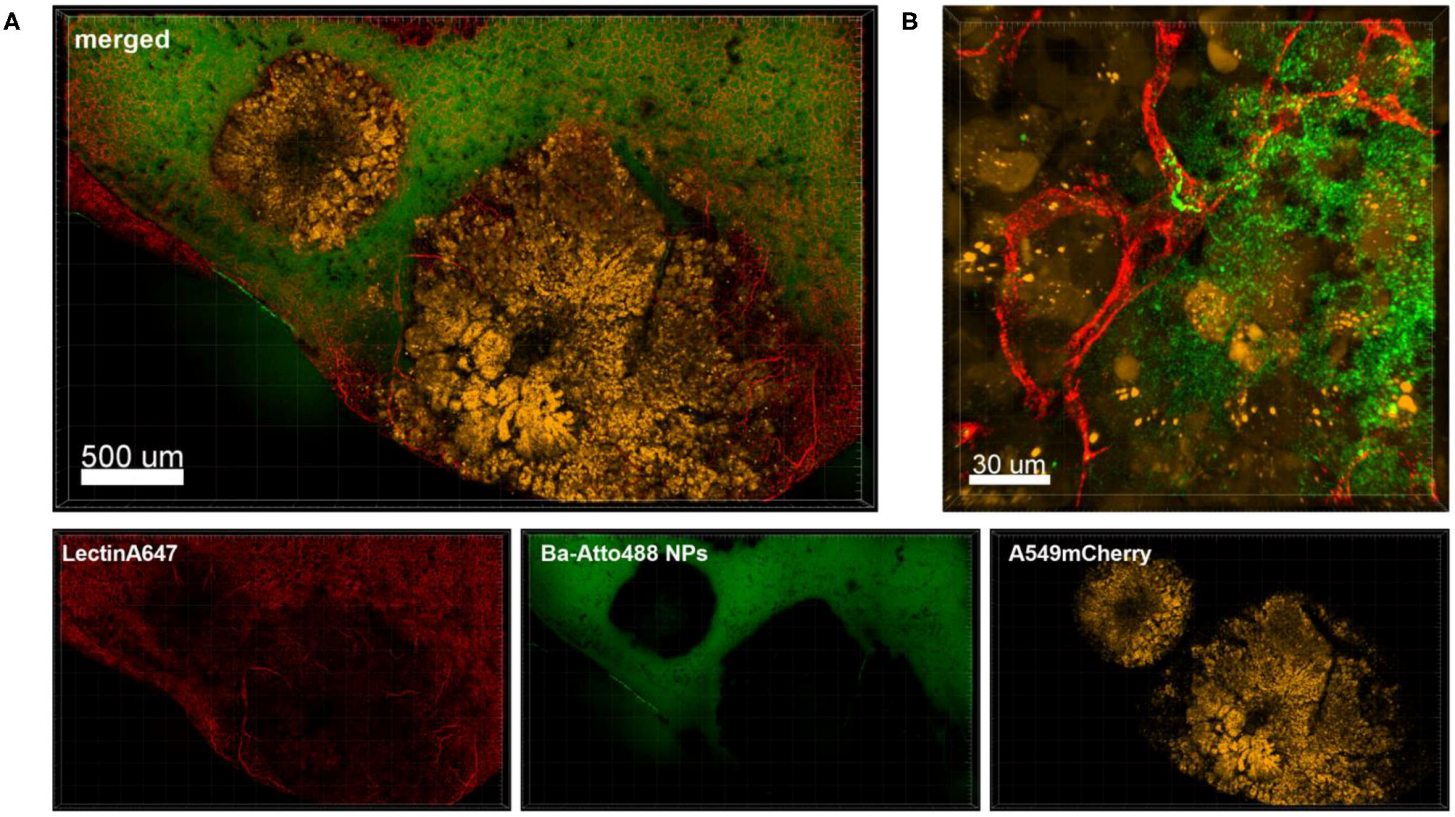
Penetration of Ba-Atto488-NPs into the tumor tissue. Representative confocal images of a lung lobe from a Nu/Nu mouse with A549-mCherry (yellow) lung tumor nodules. Lectin-A647 (red) for blood vessel staining and Ba-Atto488 NPs (green) were injected i.v. shortly before sacrifice. **(A)** Overview of two tumor nodules in the lung with central necrotic areas and poor vascularization. Images show the overlay (merged) of Lectin-A647 (red), Ba-Atto488 NPs (green), and A549-mCherry cells (yellow). Scale bar represents 500 μm. **(B)** shows a magnified region at the border of the tumor represented in **(A)**, with a leaky vessel within one of the nodules and Ba-Atto488-NPs that entered the surrounding tumor tissue in close proximity to the vessel. Ba-Atto488 NPs were detected with Airyscan technology. Scale bar represents 30 μm.

Figure 6 shows an overview of part of a lung lobe with several A549-mCherry induced tumor nodules and Ba-Atto488 NPs as well as Lectin-A647 injected shortly before sacrifice. The tumor nodules displayed a mixed intensity of mCherry fluorescent protein, which may be either due to different levels of mCherry expression or heterogeneous development of the tumor cells (Figure 6A, *yellow signals*). Furthermore, dark areas were visible in the center of the nodules, suggestive of necrosis (Figure 6A). The nodules are generally poorly vascularized as seen by a lack of bound Lectin-A647 (Figure 6A, *red signals*). This led to a very limited distribution of Ba-Atto488 NPs in the tumor nodules when compared to the healthy lung tissue, where the NPs were generally well-dispersed throughout. Some lectin-stained vessels are present at the border of the tumor and these are partly leaky, which is demonstrated by an accumulation of NPs in the surrounding tumor tissue (Figure 6B).

These results confirm earlier problems with the specific delivery of nanoparticulate drugs to tumor sites. Despite the fact that nanoencapsulated drugs have been shown preclinically to accumulate in tumors via the enhanced permeability and retention effect (EPR) (Sanna and Sechi, 2020) as well as attempts to actively target the tumors by functionalization of nanotherapies with specific antibodies, most efforts showed a very large intra- and inter-individual heterogeneity, explaining the mixed response of patients to the therapies (Dasgupta et al., 2020). It appears that tumors show vast differences in EPR-contributing parameters, such as vessel density, perfusion and permeability, tumor stroma composition and lymphatic vessel functionality (Dasgupta et al., 2020) and these differences largely contribute to the heterogenic response of tumors to nanotherapies. Our approach vividly depicts this problem by showing that the limited accumulation of NPs at the tumor site can be related to both a lack in vascularization as well as a dense stroma capsule around the tumor, offering an exemplary explanation for the frequently poor efficacy found when such strategies are implemented. Moreover, the delivery of NPs to the tumor and lymph nodes, the elimination pathway as well as their intracellular processing is dependent on the size and shape of NPs (Stylianopoulos, 2013). A fine balance has to be found between NP sizes that are large enough to avoid a rapid renal clearance, but small enough to penetrate the leaky tumor vessel pores. The *ex vivo* live cell imaging method may therefore be a useful tool for the evaluation of differently sized and composed NPs and the effect these parameters have on EPR, lymphatic drainage and modification of the extracellular matrix (Fernandes et al., 2018; Wang et al., 2019).

**Figure 7:**
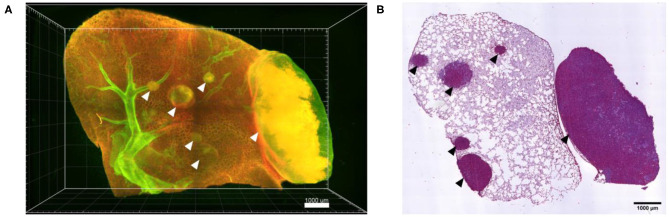
*Ex vivo* light sheet microscopy and histology is possible after live cell imaging. **(A)** Maximum projection of a 3D stitched mosaic acquired by light sheet microscopy of part of the lung lobe shown in Figure 5, displaying several A549-mCherry induced tumor nodules deep inside the lung lobe (arrow heads). **(B)** H&E staining of a paraffin section from the lung lobe displayed in **(A)** following paraffin embedding of the lung lobe and serial sectioning. Scale bars represent 1,000 μm.

Following confocal live cell visualization, the tissues can be prepared for all other common *ex vivo* visualization procedures, including light sheet microscopy and histology, as shown in Figure 7. Light sheet microscopy, which uses cleared fixed tissue revealed additional tumor nodules deep within the same lung lobe, which were not identified by confocal microscopy due to limited optical penetration. H&E staining of a 2D paraffin section from the same lung tissue confirmed several locations of tumor lesions. Combined, *ex vivo* live cell confocal imaging of entire lung lobes is thus a good preclinical method of pre-assessing the accumulation of NP-based drugs at the desired site of action, in particular in the case of tumor masses.

### Tumor Cell Dynamics

**Figure 8:**
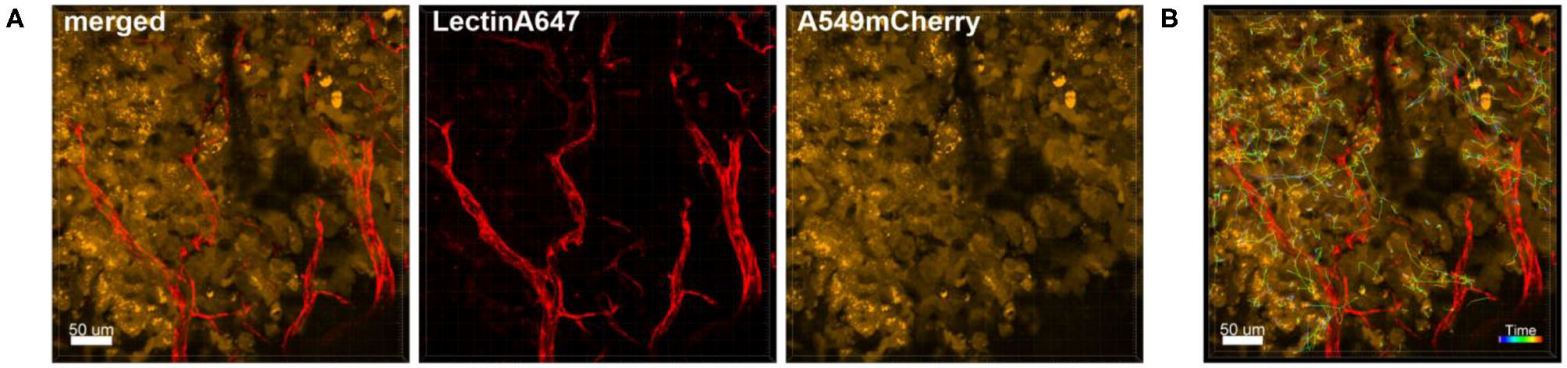
Tracking of the dynamic movement of tumor cells in the tumor mass. Confocal images of a lung lobe from a Nu/Nu mouse with A549-mCherry cell-induced tumors (yellow). Blood vessels were stained by i.v. injection of Lectin-A647 (red) shortly before sacrifice of the animal. **(A)** Images show the overlay (merged) of Lectin-A647 (red) and A549-mCherry cells (yellow). **(B)** Representative image of a time series over 1 h 34 min showing the trajectory of individual motile tumor cells. Scale bars represent 50 μm.

Also the behavior of individual or clusters of cells within a tumor mass can be studied. We have for instance observed astonishingly strong movement of tumor cells in already established tumor masses that were induced several weeks before imaging (Figure 8 and Supplementary Movie 4). This suggests that tumors are extremely active structures with dynamic cell displacement.

**Figure 9:**
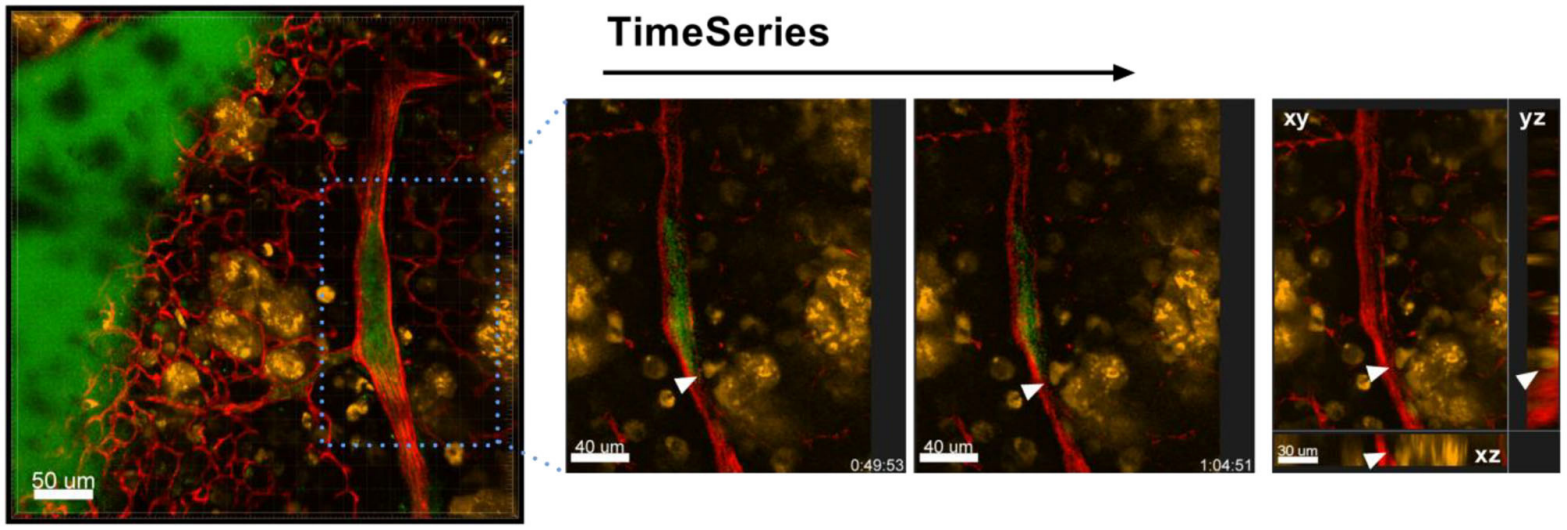
Intravasation of tumor cells into blood vessels. Left: Representative image of a time series over 1 h 4 min taken of a lung lobe from a Nu/Nu mouse with A549-mCherry cell-induced tumors (yellow). Blood vessels were stained by i.v. injection of Lectin-A647 (red) shortly before sacrifice of the animal. The three images in the right panel are zoomed in images of the boxed location in the left image. The far right image shows an orthogonal projection at the end of the time series. Scale bars represent 40 μm, overview image 50 μm.

The process of extra- and intravasation of tumor cells into and from blood vessels are also processes that could be studied in detail by *ex vivo* lung live cell imaging, an example of which can be seen in Figure 9. Imaging of the direct crossing of the tumor vascular barrier through intercellular gaps may be achieved by Airyscan technology.

### Exchange of Nanomaterial Between Tumor Cells

**Figure 10:**
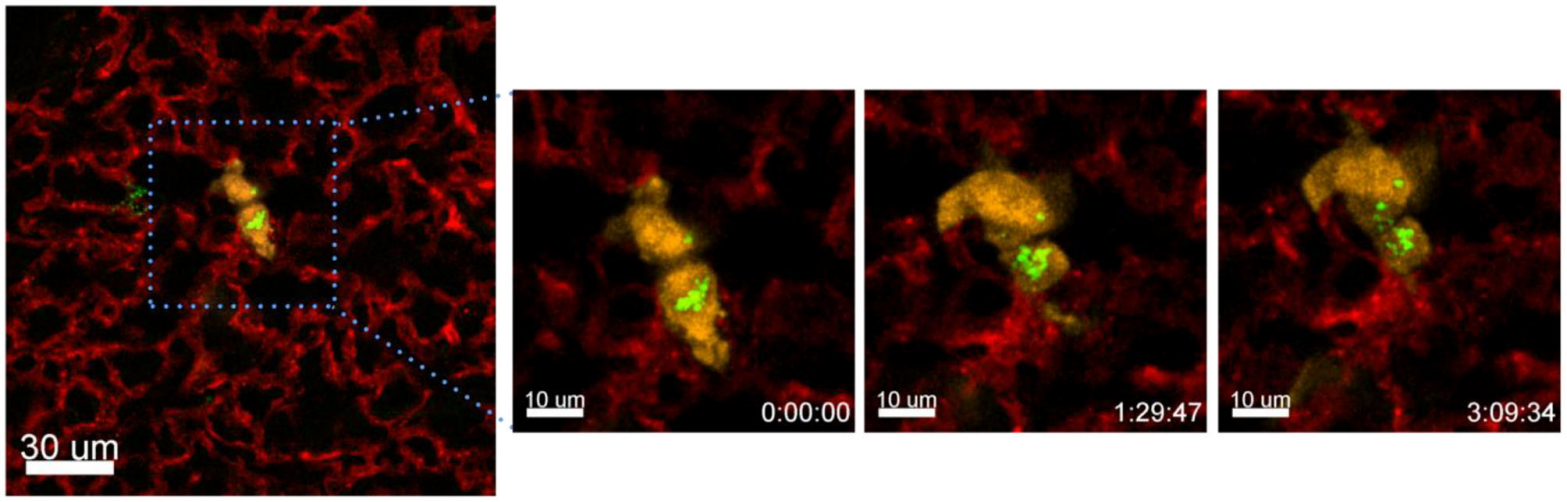
Tracing intercellular exchange by NPs. Confocal images of a lung lobe from a NMRI-Fox1nu/nu mouse, i.v. injected with Lectin-A647 (red) and Ba-Atto488 NPs (green) loaded A549-mCherry cells (yellow) shortly before sacrifice. The three images in the right panel show representative magnified images of a time series over 3 h taken from the area marked in the left image. Ba-Atto488 NPs are transferred between two tumor cells. Scale bars represent 10 μm, overview image 20 μm.

Another process that we observed with the presented technique is the exchange of material between different cells. In the following example we have incubated A549-mCherry cells with Ba-Atto488 overnight and then injected them i.v. in a NMRI-Fox1nu/nu mouse. Figure 10 and Supplementary Movie 5 clearly show the exchange of Ba-NPs between two tumor cells active in the lung. The exchange of nanomaterial took place over 3 h. As we have labeled the A549-mCherry cells with Ba-Atto488-NPs before injecting them i.v., it is most likely that the NPs are already located in late endosomes/lysosomes, which can be then transferred by extracellular shedding. Fluorescent NPs are of advantage here for the visualization of this process.

Intercellular organelle and EV exchange has been described many times, but most have only been shown in cell culture (Rogers and Bhattacharya, 2013). Moreover, the transfer of exosomes from one cell to another is a common feature especially in immune cells. EVs are vehicles for bidirectional communication between cells and carry bioactive molecular cargoes, including proteins, lipids and nucleic acids that can affect the functions and phenotypes of recipient cells by altering gene expression or by activating various signaling pathways (Maacha et al., 2019). EVs can carry their cargo from the parent cell and can be captured by neighboring or distant recipient cells through the interaction of vesicular ligands with cellular receptors, but the precise mechanisms of interaction remain poorly characterized. Tumor-derived EVs may contain tumor-specific antigens on their surface or miRNAs and brings an advantage to cells that can share this material with other cells. Studies suggest that vesicles can be internalized and could fuse with the recipient cell either at the plasma membrane or after internalization (Théry et al., 2009). While we cannot directly show that the transfer of NPs happened through EVs, we speculate that this is the most likely process. Importantly, to our knowledge this is the first description of material exchange between tumor cells in live lung tissue imaging.

### Shedding of Membrane Vesicles

**Figure 11:**
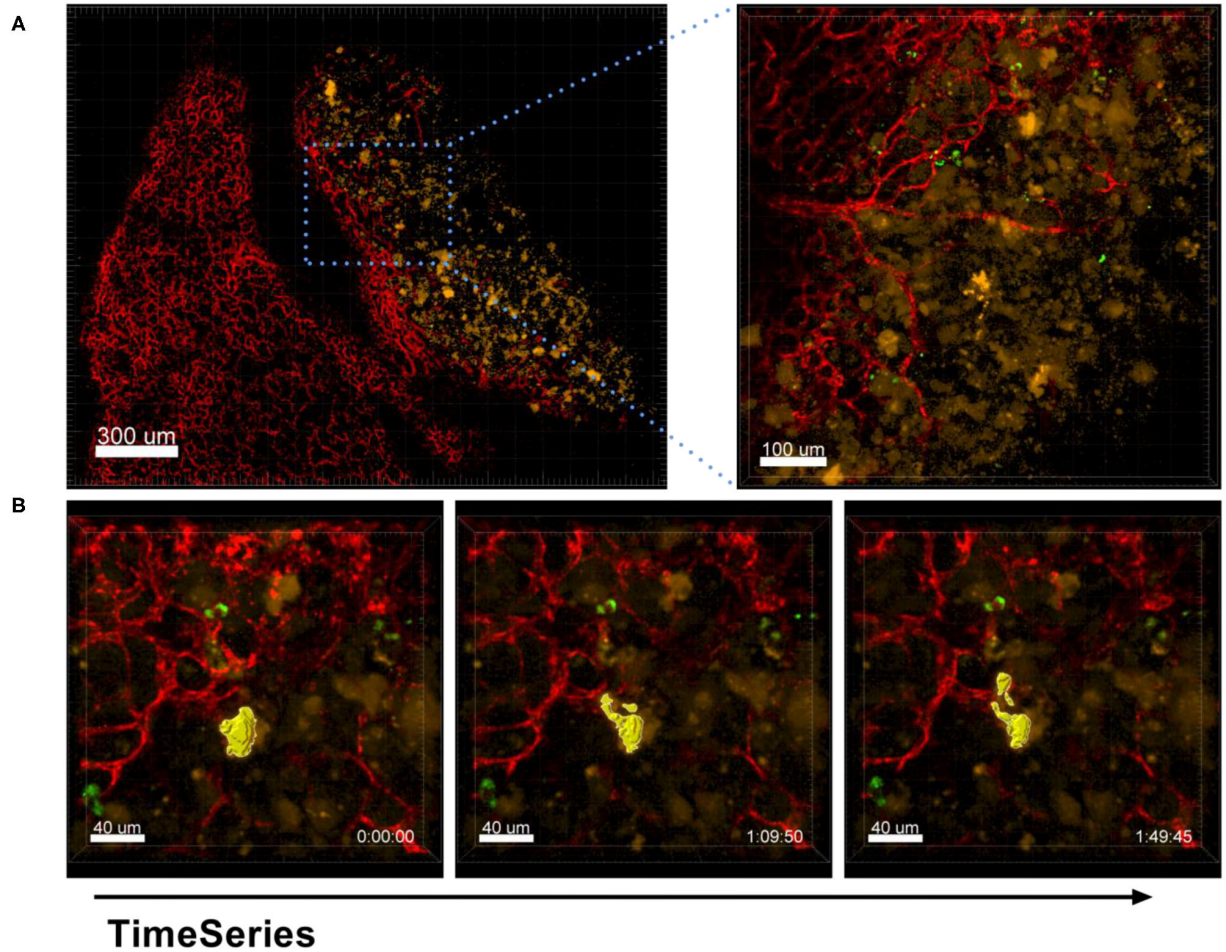
Shedding of EVs. Confocal microscopy images of a lung lobe from a Nu/Nu mouse with a A549-mCherry tumor (yellow) and Lectin-A647 (red) and Ba-Atto488-NPs injected i.v. 5 min before sacrifice of the mouse. **(A)** Overview image showing a tumor nodule (yellow) and vascularization (red). Scale bars represent 100 μm, overview image 300 μm. **(B)** Representative images from a time series over 1 h 50 min with a frame interval of 180 s. Scale bars represent 40 μm.

The process of EV production by tumor cells was also captured by our *ex vivo* live cell microscopy approach. As shown in Figure 11 and Supplementary Movie 6A, A549-mCherry tumor cells from an established tumor nodule were observed in the process of producing vesicles of about 10 μm in size and releasing them in a blebbing manner.

A second example of EVs shedding of similar size can be seen in Supplementary Movie 6B, which was produced shortly after i.v. injection of A549-mCherry cells. This movie also shows that ex-vivo live cell imaging visualized the active movement of tumor cells within a blood vessel over about 3 h 19 min hours. Furthermore, several tumor cells were observed that undergo apoptosis, or others seem to be more resistant and release EVs and rapidly explore the surrounding tissue. The lung is the organ with the highest vascular density in humans and thus substantially contributes to the circulation of EVs, lipid bilayer-delimited particles that contribute to the development of metastasis. Exosomes, microvesicles and oncosomes secreted by tumors have been shown to inhibit immune responses, but in conjunction with adjuvants they can also induce potent antitumor responses (Théry et al., 2009). Moreover, all these EVs have been shown to be key players in signaling of tumor cells over long distances, contributing to the promotion of a pre-metastatic niche and reprogramming of the stroma (Minciacchi et al., 2017; Wortzel et al., 2019). While exosomes may be too small (up to 150 nm) to visualize by our described approach, microvesicles (100–1,000 nm) and oncosomes (1–10 μm) are in the range of good visibility by confocal microscopy. It is therefore probable that the EVs we detected by *ex vivo* live cell imaging are oncosomes. Thus, this method may support the better understanding of EVs and their distinctive roles and may also contribute to the clarification of the controversy that exists to date on the nomenclature of EVs (Meehan et al., 2016; Witwer and Théry, 2019). This method is thus a valuable tool to identify the continuous formation of EVs by the tumor cells, which most of the time cannot be detected by slice imaging methodologies once that the EVs lose the expression of key cancer biomarkers used to identify the tumor cells.

## Conclusion and Potential

*Ex vivo* live cell confocal microscopy provides a new way of studying the interactions of nanoparticles with immune and tumor cells and the vessel endothelium. As we show by several examples in the mouse lung, the method presents a notable alternative to more demanding techniques such as intravital lung microscopy using windows and to *in vivo* optical imaging that provide substantially lower resolution. It is mostly suitable for the visualization of processes that occur within the vessel/tumor interfaces, such as delivery of NPs to the tumor, interaction of NPs with blood monocytes, intra- and extravasation. Thus, the method may support the characterization of nanoparticulate therapies, studying the dependence of NP uptake on the heterogeneity and microenvironmental features of tumors as well as the optimization of NPs size and functionalization for specific targeting. By engineering specific NP surfaces it should in theory be possible to modulate and control host responses. Such processes may also be studied by this technique. While we are using fluorescently tagged cell lines and transgenic mice to visualize specific cells, an alternative or additional approach could be the injection of fluorescent antibodies before sacrifice and imaging of the lung. Moreover, the approach is able to supply new information on cell processes that are not directly and instantaneously affected by a loss of air flow or breathing mechanics, such as the behavior of tumor masses in the lung, including the production of extracellular vesicles and their fate, intercellular exchange of material/organelles and the processing of tumor material by immune cells. While we focus on interactions with nanoparticles, this method may provide novel information on a number of other cellular events, such as the phagocytosis of tumor cells by macrophages and other cells; the immediate cellular effects of treatments such as chemotherapies, including the live visualization of apoptotic events; the immediate reaction of immune cells at encounter of allergens and real-time detection of intracellular and metabolic processes in live cells.

## Data Availability Statement

All datasets generated for this study are included in the article/Supplementary Material.

## Ethics Statement

The animal study was reviewed and approved by Nds. Landesamt für Verbraucherschutz und Lebensmittelsicherheit.

## Author Contributions

FR-G conceived and designed the experiments, acquired and analyzed the data, and wrote the paper. NF acquired and analyzed the light sheet microscopy data. AK designed and prepared the nanoparticles. FA conceived the experiments and contributed to writing the paper. MM conceived, designed, prepared the experiments, and wrote the paper. All authors contributed to the article and approved the submitted version.

## Conflict of Interest

AK was employed by nanoPET Pharma GmbH. The remaining authors declare that the research was conducted in the absence of any commercial or financial relationships that could be construed as a potential conflict of interest.
