# Supplementary figures and images for: Ex vivo Live Cell Imaging of Nanoparticle-Cell Interactions in the Mouse Lung
Source: Front Bioeng Biotechnol. 2020 Oct 30;8:588922. doi: 10.3389/fbioe.2020.588922 (PMC7661932; doi:10.3389/fbioe.2020.588922)

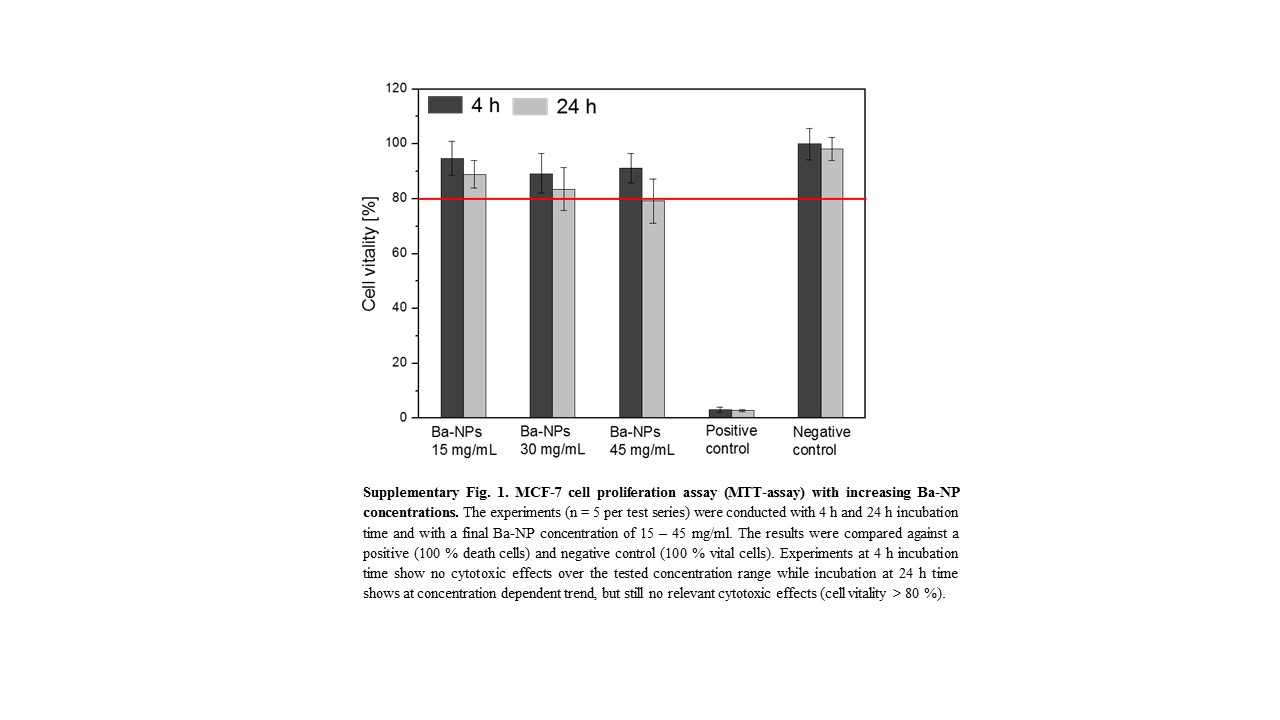

Supplement: Supplementary file 2 [file Image_1.JPEG]
